# Pulmonary adenocarcinoma mimicking miliary tuberculosis in a 20‐year‐old man: A clinical case report

**DOI:** 10.1002/ccr3.7028

**Published:** 2023-03-02

**Authors:** Arash Seifi, Nahid Shafiee, Maryam Moradi

**Affiliations:** ^1^ Department of infectious diseases, Imam Khomeini Hospital Tehran University of Medical Sciences Tehran Iran; ^2^ Eye Research Center, The Five Senses Health Institute, Rassoul Akram Hospital Iran University of Medical Sciences Tehran Iran

**Keywords:** lung adenocarcinoma, lung cancer, miliary nodule, miliary tuberculosis

## Abstract

Lung adenocarcinoma (LA) is the most common subtype of lung cancer with nonsignificant manifestations. Some benign conditions can mimic LA in symptoms and even chest imaging.

In this case report, we are discussing a young man without any significant medical history with metastatic LA, initially presumed military TB.

## Graphical abstract

Various conditions can present a miliary view in chest imaging, including benign conditions such as bacterial and fungal infections and occupational disorders. More severe cases can appear the same, such as miliary TB and metastatic lung cancers, especially adenocarcinoma. Lung adenocarcinoma (LA) is the most frequent histological subtype of lung cancer worldwide. Early diagnosis and proper treatment are the key points in patients’ survival.

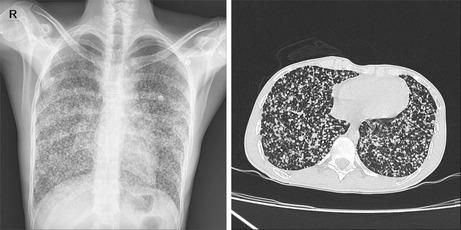

## 1 BACKGROUND

Lung adenocarcinoma (LA) is the most frequent histological subtype of lung cancer worldwide. This condition is categorized from preinvasive lesions to metastatic adenocarcinoma.^1^, ^2^ Smoking is the most common responsible risk factor for LA so far, along with a family history of lung cancer, occupational exposure, and genetic mutations. In the early stages, LA can be asymptomatic while in the late stages, non‐specific symptoms such as coughing, weight loss, and hemoptysis can occur.^3^

Miliary tuberculosis (TB) is the wide‐spreading dissemination of *Mycobacterium tuberculosis* through the bloodstream. It can affect single or several organs and through a hematogenous way to the brain.^4^ Miliary TB can present with nonspecific manifestations, including fever, weight loss, sweats, and anorexia mimicking other diseases, especially primary and secondary lung cancers. Immune suppression due to cancer, HIV infection, malnutrition, diabetes, transplantation, and end‐stage renal disease are risk factors for this condition.^5^

The miliary pattern in lung imaging can present in miliary TB, occupational lung diseases, different fungal infections, sarcoidosis, and metastatic diseases from primary or secondary lung cancers; therefore, diagnosis cannot be distinguished by CT imaging alone. Further investigations such as serological and pathological tests are necessary for a diagnosis.^6^, ^7^

In the current article, we are presenting a young man with no prior medical history or risk factor with metastatic lung adenocarcinoma mimicking miliary TB in imaging.

## 2 CASE PRESENTATION

A 20‐year‐old man with no significant medical history visited a tertiary referral collegiate hospital in Tehran on 5 June 2022, with severe headache (frontal, nonpositional, pulsatile without any photophobia, phonophobia, and visual disturbances), nausea, vomiting, malaise, nonbloody sputum for 2 weeks, and significant weight loss of about 15 kg in recent 2 months. His family history was negative for TB or lung malignancies. At admission, his vital signs were in the normal range. On physical examination, his conjunctiva was pale along with cervical and supraclavicular lymphadenopathies. Other neurological and non‐neurological examinations were normal.

**FIGURE 1:**
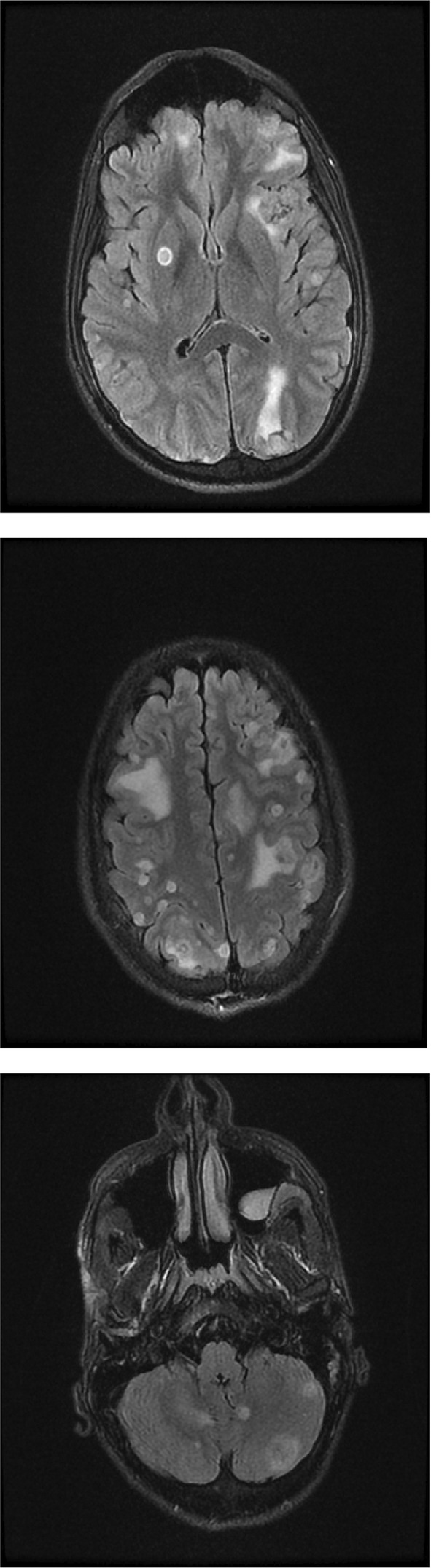
Patient's brain magnetic resonance image.

Due to the severe headache, which lasts for 30 min each time and repeats every 3 h, neurological consultation was done. MRI of the brain demonstrated multiple hyperdense lesions were seen (Figure 1).

To rule out endocarditis, echocardiography was done, which detected severe pericardial effusion of about 750 CCs, and pericardiocentesis along with a myocardial biopsy and pericardial smear and culture has been done.

**FIGURE 2:**
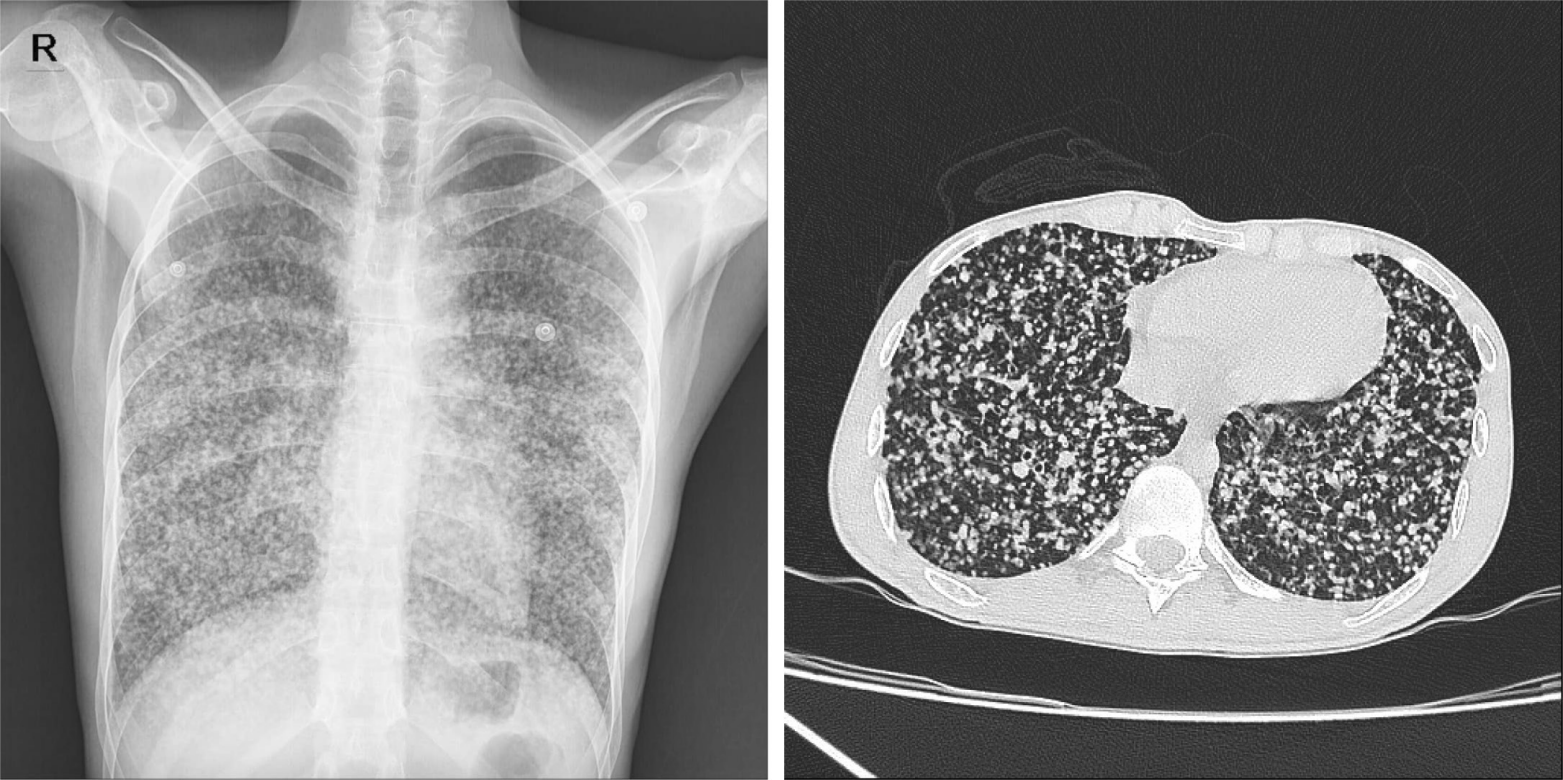
Patient's lung computed tomography.

In abdominal and chest spiral CT scans, lesions suspected of miliary TB, including adrenal involvement, multiple pulmonary small nodules, and mediastinal lymphadenopathies were seen (Figure 2). In infectious disease consultation sputum smear and culture for *Mycobacterium tuberculosis*, blood and sputum MTB‐PCR, cytology MTB‐PCR, technetium‐99 m‐ethambutol (Tc‐EMB) scintigraphy, and IGRA were sent.

Sputum smear and culture, blood, and sputum MTB‐PCR were negative, but Tc‐EMB scintigraphy was positive. According to the patient's clinical condition and positive scintigraphy, it was decided to prescribe an anti‐TB regimen including isoniazid, rifampin, pyrazinamide, and ethambutol along with dexamethasone.

Following the results of the pericardial and myocardial biopsy, the patient was diagnosed with lung adenocarcinoma with massive intra‐lung and remote metastasis. According to the new diagnosis, the anti‐TB regimen was stopped and chemotherapy was started for the patient. Chemotherapy course consists of Bevacizumab (Stivant) and Zoledronic acid (Zobis) along with Dexamethasone injection was used every 2 weeks, and for brain metastasis, whole‐brain radiation therapy (WBRT) was done.

Due to the rarity of the diagnosis in our young patient without any prior risk factor, an immunological study was done, which was all negative. The patient's clinical condition starts to improve after the chemotherapy, and he is alive at the moment of writing this article.

## 3 DISCUSSION

Lung cancer is the most common cancer diagnosis worldwide, with a high rate of mortality. Lung adenocarcinoma, a non‐small‐cell lung cancer is the most common histological subtype with nonspecific symptoms and outcomes. Early diagnosis and treatment are critical in patients' outcomes and survival.^8^

Miliary TB is a lethal state of tuberculosis in immunosuppressed patients with widespread dissemination of *Mycobacterium* even in the brain. Various symptoms can be present due to the site of involvement. Diagnosis is complicated due to the nonspecific radiograph findings.^9^

Chest CT scan is a critical tool in LA diagnosis, while it can appear differently, mimicking nonmalignant and infectious diseases.^10^ Miliary shadows on imaging can appear in different diagnoses, especially in infectious diseases such as miliary TB and histoplasmosis. Metastatic primary and secondary lung cancers can also present as miliary nodules.^11^

In TB endemic areas such as our country, it is always critical to treat patients with nonspecific symptoms and miliary shadows on chest imaging as miliary TB cases, while further evaluations are necessary for the proper diagnosis. Miliary nodules in chest imaging are a rare finding in LA, while probable. We have to consider LA as an important differential diagnosis in those with miliary shadows because it has a bad prognosis in late diagnosis. Like our patient, several cases have been reported to be LA, while their initial diagnosis was miliary TB.^7^, ^11^, ^12^

## 4 CONCLUSION

Various conditions can present a miliary view in chest imaging, including benign conditions such as bacterial and fungal infections and occupational disorders. More severe cases can appear the same, such as miliary TB and metastatic lung cancers, especially adenocarcinoma. Early diagnosis and proper treatment are the key points in patients' survival.

## AUTHOR CONTRIBUTIONS

**Arash Seifi:** Conceptualization; supervision. **Nahid Shafiee:** Data curation; resources.

## FUNDING INFORMATION

None.

## CONFLICT OF INTEREST STATEMENT

We have no conflict of interest to declare.

## CONSENT

Written informed consent was obtained from the patient to publish this report in accordance with the journal's patient consent policy.

## Data Availability

Data sharing is not applicable to this article as no datasets were generated or analyzed during the current study.
